# Microvalve with Trapezoid-Shaped Cross-Section for Deep Microchannels

**DOI:** 10.3390/mi12111403

**Published:** 2021-11-15

**Authors:** Maho Kaminaga, Tadashi Ishida, Toru Omata

**Affiliations:** 1 Department of Mechanical Engineering, National Institute of Technology, Toyota College, 2-1 Eiseicho, Toyota 471-0067, Japan; 2 Department of Mechanical Engineering, School of Engineering, Tokyo Institute of Technology, 12-1 Ookayama, Tokyo 152-8550, Japan; ishida.t.ai@m.titech.ac.jp (T.I.); omata.t.aa@m.titech.ac.jp (T.O.)

**Keywords:** microfluidic device, pneumatic microvalve, inclined lithography

## Abstract

Simple microfluidic systems for handling large particles such as three-dimensional (3D) cultured cells, microcapsules, and animalcules have contributed to the advancement of biology. However, obtaining a highly integrated microfluidic device for handling large particles is difficult because there are no suitable microvalves for deep microchannels. Therefore, this study proposes a microvalve with a trapezoid-shaped cross-section to close a deep microchannel. The proposed microvalve can close a 350 μm deep microchannel, which is suitable for handling hundreds of micrometer-scale particles. A double-inclined lithography process was used to fabricate the trapezoid-shaped cross-section. The microvalve was fabricated by bonding three polydimethylsiloxane layers: a trapezoid-shaped liquid channel layer, a membrane, and a pneumatic channel layer. The pneumatic balloon, consisting of the membrane and the pneumatic channel, was located beneath a trapezoid-shaped cross-section microchannel. The valve was operated by the application of pneumatic pressure to the pneumatic channel. We experimentally confirmed that the expansion of the pneumatic balloon could close the 350 μm deep microchannel.

## 1. Introduction

A variety of microfluidic devices, ranging from simple disposable microchannels to highly integrated microfluidic systems, have contributed to the advancement of biology [1]. These microfluidic devices have been developed for handling small particles (nanoscale) to middle-size particles (microscale), such as biomolecules and cells. In experiments with biomolecules, these devices have been applied to polymerase chain reactions (PCRs) [2], DNA analysis and sequencing [3,4], protein isolation [5,6], and immunoassays [7]. In the field of cell analysis, they have been applied to cytotoxicity assays [8,9], co-culture of multiple cell-types [10,11], and screening of target-cell-specific binding molecules [12]. Microfluidic devices with deep microchannels and large cross-sectional areas have also been developed [13,14] to handle large particles (on the scale of hundreds of microns ), such as 3D cultured cells, microcapsules, and animalcules. For example, 3D cultured cells must have a diameter of 200 μm to mimic a tumor state [15]. The microcapsules containing the animal cells have a 100–300 μm diameter [16,17]. In addition, the maximum size of water bottle plankton is about 200 μm [18], which is therefore the required channel depth for handling large particles.

Microvalves are essential components in the development of highly integrated microfluidic devices because they are required for precise fluidic control [19]. Accordingly, a microvalve that can close a microchannel for handling large particles must be developed. Among the various types of microvalves that are integrated with microfluidic devices [20], pneumatic microvalves are suitable for biological experiments. Pneumatic microvalves consist of two microchannels and a thin membrane between the microchannels [21]. The microchannels and the membrane are made of polydimethylsiloxane (PDMS). The thin membrane bends when the pneumatic pressure is applied to one of the pneumatic channel, and causes the other microchannel to be closed [22]. This type of microvalve allows the use of scarce biogenic samples without waste because there is no dead volume. In addition, they also have advantages in biological experiments, including ease of sterilization, rapid response, simple configuration, and compactness.

**Figure 1:**
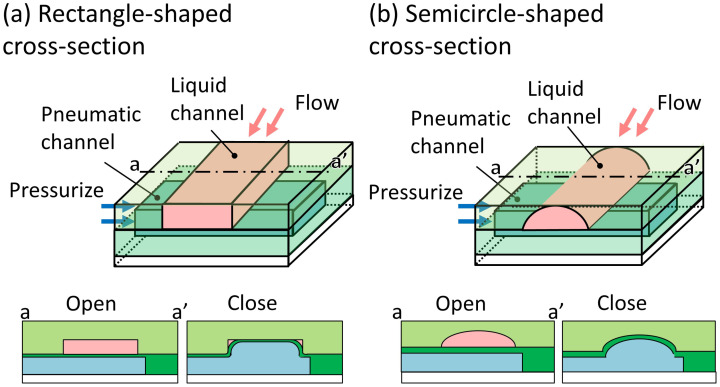
Conventional cross sections of microchannels of pneumatic pressure-driven microvalves. (**a**) Rectangle-shaped cross-section; (**b**) semicircle-shaped cross-section [26].

Photolithography, which is commonly used for fabricating pneumatic microvalves, has the advantageous capability of incorporating fine microstructures and producing excellent surface properties. However, a typical microchannel with a rectangle-shaped cross-section (Figure 1a), a replica of a photoresist mold, cannot completely seal its inner angles to liquid flow. If there is a gap at the inner angles, single cells detached from the 3D cultured cells [23] will pass through the valve, resulting in cross-contamination. Semicircle-shaped cross-sectional microchannel, which can be sealed completely (Figure 1b), is fabricated by using reflow process [24]. However, the maximum depth of the microchannel fabricated by using the reflow process is 65 μm [25], which is not suitable for microvalves requiring deep microchannels. Although semicircle-shaped cross-sectional microchannels can be fabricated by using 3D-printer, the rough surface of the microchannel is not suitable for observation under the microscope. Obtaining a highly integrated microfluidic device for handling large particles is difficult because there are no suitable microvalves for deep microchannels.

For these reasons, pneumatic microvalves have been developed for closing microchannels that are hundreds of micrometers deep. For example, microvalves with vertical membrane could close a deep microchannel [27]. The valve was fabricated using double-sided molding. Although this microvalve is capable of closing a deep microchannel, the 3D microchannel configurations are too complex for integrated microfluidic system. A microvalve for a microchannel with a deep semicircle-shaped cross-section is fabricated using grayscale lithography on SU-8 (thick negative photoresist) [28]. However, an expensive digital micro-mirror device is required for this technique. Thus, to prevent such costs, a relatively simple and inexpensive technique is required. In the previous study, we used a single inclined lithography [29] process to fabricate a pneumatic microvalve that enables complete closure of a deep microchannel [26]. The cross-section of the microchannel was parallelogram-shaped. However, the maximum microchannel depth attained was 100 μm [26], which is insufficient for handling large particles.

Therefore, the proposal of this paper is to develop a simple pneumatic microvalve for closing a microchannel deeper than 200 μm. We propose a pneumatic microvalve with a trapezoid-shaped cross-section. The double-inclined lithography process was used to fabricate a trapezoid-shaped cross-section. The process does not require expensive device or complicated method. The performance of the microvalve was evaluated by flowing spheroid suspension and microbead.

## 2. Material and Method

### 2.1. Working Principle of the Trapezoid-Shaped Cross-Section Microvalve

**Figure 2:**
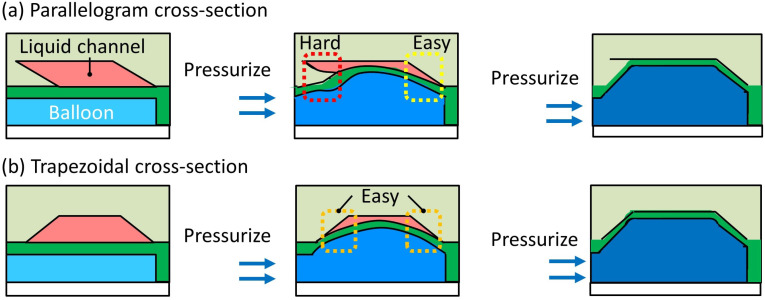
Cross-sections of microchannels of pneumatic pressure-driven microvalves. (**a**) Parallelogram-shaped cross-section; (**b**) trapezoid-shaped cross-section.

The maximum closing depth of the valve’s microchannel with the parallelogram-shaped cross-section was 100 μm [26]. It requires a significantly high operating pressure to seal a deeper microchannel, even if the valve can operate without leakage at that operating pressure. This is because the overhang at the acute angle is thicker and thus more difficult to bend in accordance with the depth of the microchannel (Figure 2a). Therefore, we propose a trapezoid-shaped cross-section, as shown in Figure 2b. In the trapezoid-shaped cross-section, the inner angles of both sides are obtuse; thus, the microchannel can be sealed only by deformation of the membrane.

### 2.2. Fabrication

#### 2.2.1. Design

**Figure 3:**
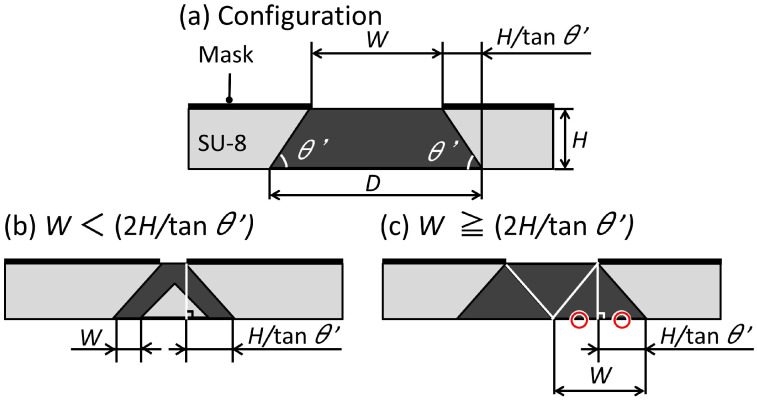
Schematic illustration of the inclined lithography for making a trapezoid-shaped cross-section.

Figure 3a shows a schematic illustration of the trapezoid-shaped cross-section and the inclined lithography mask. The upper base length of the trapezoid-shaped cross-section is similar to the mask aperture width *W*. However, the lower base length *D* is different from *W*. When the depth of the trapezoid-shaped cross-section is *H* and the bottom angle of the trapezoid-shaped cross-section is $\theta^{\prime }$, the relationship between *W* and *D* is given by the Equation (1) below.

(1) $$D=W+2H/\tan\theta^{\prime }$$

As shown in Figure 3b, when *W* is shorter than 2*H*/tan$\theta^{\prime }$, some areas are not fully exposed by double exposure. As shown in Figure 3c, when *W* is longer than 2*H*/tan$\theta^{\prime }$, the entire trapezoid-shaped cross-section is doubly exposed. Simply, the minimum value of *D* of the trapezoid-shaped cross-section that can be fabricated with only double inclined exposure is 4*H*/tan$\theta^{\prime }$. The angle of inclination during exposure θ and the angle $\theta^{\prime }$ of the fabricated structure can be expressed by Equation (2) below [29].

(2) $$\theta^{\prime }=90-\arcsin(\sin\theta /1.67)$$

#### 2.2.2. Fabrication Method of the Trapezoid-Shaped Cross Sectional Microvalve

The trapezoid-shaped cross-section mold can be fabricated by performing two inclined exposures [29], eliminating the need for resist reflow or grayscale lithography. Furthermore, by adopting a photolithographic process instead of cutting, a deep microchannel and microstructure such as micropillars can be fabricated in one piece.

**Figure 4:**
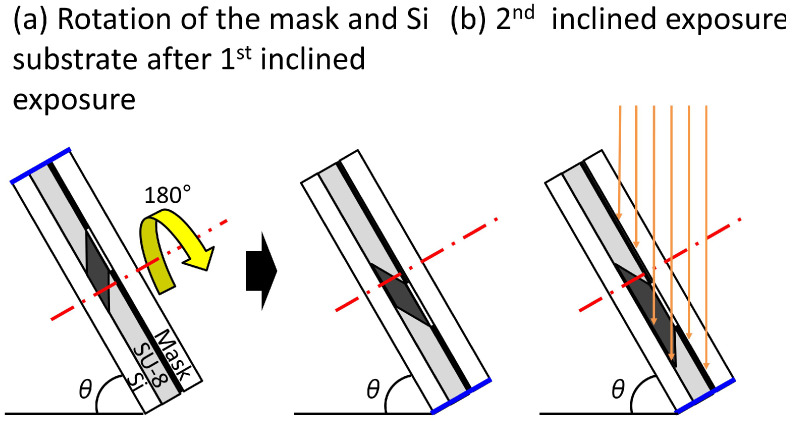
Schematic illustration of the exposure process for making a trapezoid-shaped cross-section mold. (**a**) Rotation of the mask and Si substrate after first inclined exposure; (**b**) second inclined exposure.

Figure 4 shows how the trapezoid-shaped cross-section molds are fabricated by the inclined lithography method. The mold was fabricated by changing the orientation of the substrate and repeating the inclined exposure. First, a pre-baked photoresist on a substrate is masked and fixed. Next, the mask and the substrate are inclined using a jig fabricated at a specified angle and exposed.Thereafter, the mask and the substrate are rotated at 180 degrees around the axis, as shown in Figure 4a, with the mask and the substrate fixed relatively. After the rotation and re-exposure, the cross-section of the exposed photoresist becomes trapezoidal in shape (Figure 4b). By developing the photoresist, a trapezoid-shaped cross-section mold can be fabricated.

**Figure 5:**
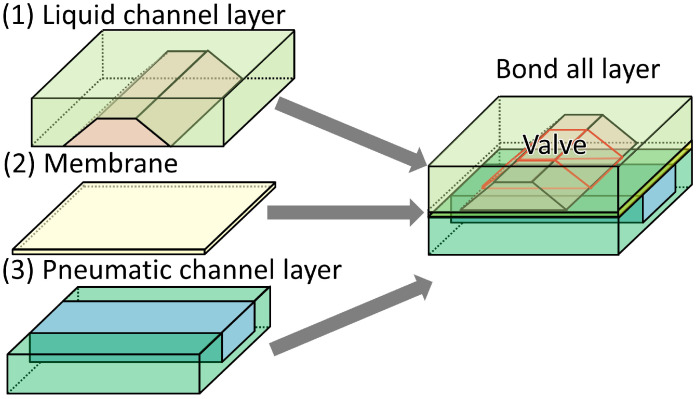
Schematic illustration of the layers of the microvalve with the trapezoid-shaped cross-section.

**Figure 6:**
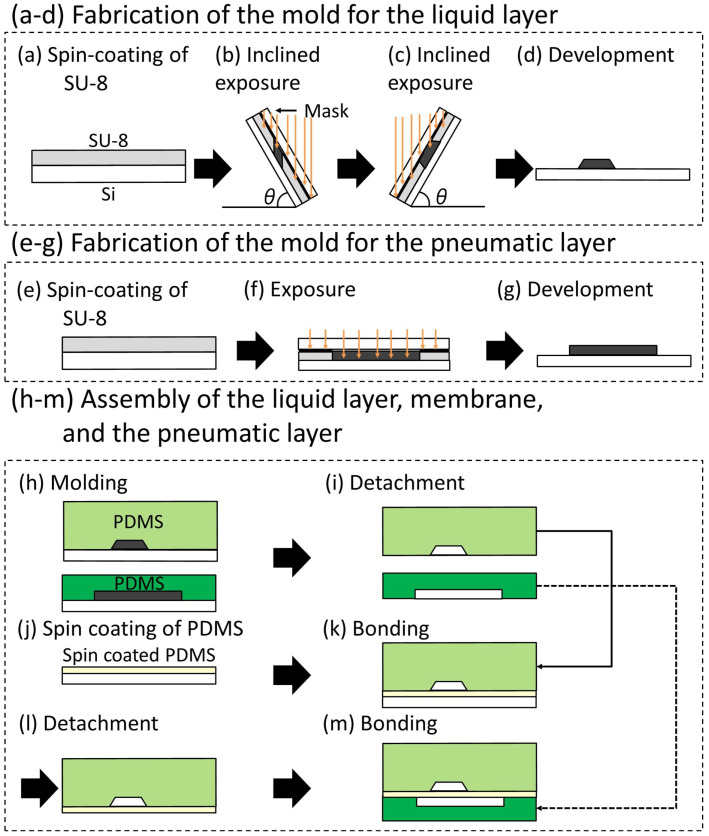
Fabrication process of the pneumatic microvalve with trapezoid-shaped cross-section liquid channel.

The microvalve consisted of three layers (Figure 5): a liquid channel, a membrane, and a pneumatic channel. The pneumatic and liquid channels were overlaid at the position of the microvalve. All the layers of the microfluidic device were fabricated by soft lithography and then bonded to each other (Figure 6). The microchannel in the liquid channel layer had a trapezoid-shaped cross-section, which was obtained by inclined photolithography.

The complete fabrication process is as follows (Figure 6): (a) SU-8 (SU-8 2150, Microchem, Westborough, MA, USA) was spin-coated on a Si substrate. (b,c) A photomask of the liquid channel was aligned on the Si substrate. The substrate with the photomask was inclined at 60 degrees and exposed to ultraviolet (UV) light. The trapezoid-shaped cross-section was fabricated by changing the orientation of the substrate and repeating the inclined exposure. (d) The unexposed SU-8 was etched away in the development process, producing the mold of the liquid channel. (e–g) The molds for the pneumatic channel were fabricated by photolithography without inclining the substrate. (h) Polydimethylsiloxane (PDMS; Silpot 184 W/C, Dow Corning Toray, Tokyo, Japan (The base polymer to curing agent ratio was 10:1 by weight)) was cast into the molds of both channels. (i) The PDMS structures for the liquid and pneumatic channel layers were detached from the molds. (j) A membrane between the liquid channel and the pneumatic channel was fabricated by spin-coating PDMS on a flat Si substrate. (k) The PDMS structure for the liquid channel layer was bonded to a thin membrane on the flat Si substrate with surface activation by vacuum UV irradiation. (l) The bonded PDMS structure was detached from the Si substrate. (m) The bonded PDMS structure was attached to the pneumatic layer with surface activation by vacuum UV irradiation.

#### 2.2.3. Fabricated Microvalve

**Figure 7:**
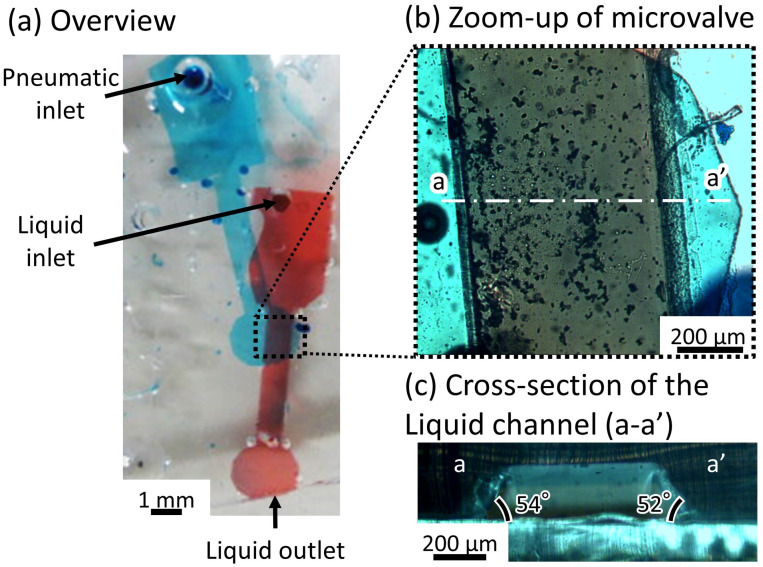
Microfluidic device with the microvalve for a tall microchannel. (**a**) Overview of the microfluidic device; (**b**) zoom-up of the microvalve; (**c**) cross-section of the liquid channel (a-a’).

Figure 7a shows the fabricated microfluidic device. The liquid and pneumatic channels were filled with red- and blue-dyed water to improve visibility. Figure 7b shows a magnified image of the valve area. Figure 7c shows the cross-sectional view of the microvalve. Blue gradation on both sides of the liquid channel represents the obtuse-angled structure. The length of the liquid channel was set to 2 mm for ease of alignment. The width at the top, bottom, and the depth of the liquid channel were 500 μm, 750 μm, and 225 μm, respectively. The diameter of the inlet was 1 mm. The thin membranes were 40 μm thick. The angle of inclination during exposure was set to 60 degrees, which is similar to that of the previously developed microvalve with a parallelogram-shaped cross-section [26]. The maximum depth of the inflated pneumatic balloon was between 30 and 40 percent of the pneumatic channel’s [30] width. To close the 225-μm deep liquid channel, the width of the pneumatic channel was set to 750 μm. To prevent the pneumatic channels and the membranes from sticking [26], the depth of the pneumatic channels and the thickness of the membrane were set to 50 μm and 40 μm, respectively.

### 2.3. Experimental Methods

#### 2.3.1. Flow Velocity and Pressure

We examined the performance of the microvalve, that is, the capability of introducing suspension of the large particles through the trapezoid-shaped cross-section channel, relationship between flow rate and velocity in the trapezoidal channel, and response time to open/close the channel using the microvalve. First, a PBS (Phosphate-buffered saline, Wako, Tokyo, Japan) droplet containing NCI-N87 cell spheroids with a diameter of 200 μm was introduced into the trapezoidal cross-sectional channel to ensure that the three-dimensional cultured cells could pass through the channel without clogging. Thereafter, to confirm the closure of the inner angle of the liquid channel, a microbead (Polybead Polystyrene Microspheres (2.5% Solids-Latex), 10.0 μm, Polyscience, PA, USA) suspension in blue-dyed water (Food dye blue, Kyoritsu-foods, Tokyo, Japan) was introduced into the microchannel while the microvalve operated. The diameter of the microbeads was 10 μm, which is the comparable size of single mammalian cells. Pneumatic pressure from 0 and 200 kPa was applied to the pneumatic channel in increments of 50 kPa. The flow rate of the bead suspension was 5 μL/min.

**Figure 8:**
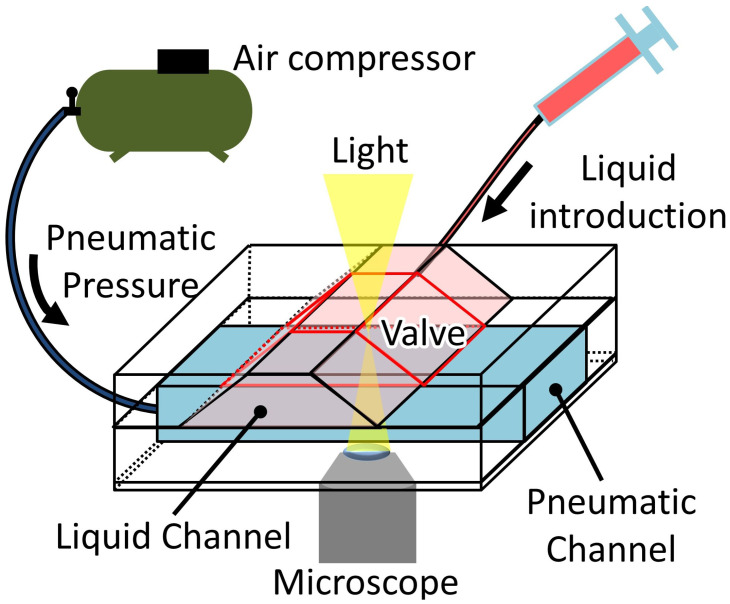
Experimental setup for the performance measurement of the microvalve.

Figure 8 shows the experimental setup for measuring the microvalve’s performance. The experiment in this section was conducted under a microscope (IX-73, Olympus, Tokyo, Japan). The bead suspension contained in a syringe connected to the liquid microchannel was introduced at a constant flow rate using a syringe pump (KDS200, KD Scientific, Holliston, MA, USA). A pneumatic pressure source (OFP-07005, Iwata, Kanagawa, Japan) was connected to the inlet of the microfluidic device’s pneumatic channel via a solenoid valve array (SY114-5LZ, SMC, Tokyo, Japan) and regulator (IR1020-01BG-A, SMC, Tokyo, Japan) to apply the necessary pressure to the pneumatic channel. All the experiments were recorded using a National Television System Committee (NTSC) video camera (30 fps). The frames extracted from the videos were analyzed using the Tracker Video Analysis and Modeling Tool (Douglas Brown, Aptos, CA, USA) to track the beads’motion.

Occasionally, the frames had afterimages of fast-flowing beads. Such fast bead flows were not analyzed because the afterimages may have reflected inaccurate speeds; however, the relatively slow bead flows with clear images were analyzed. The flow velocities of the microbeads were measured by tracking the positions of three beads selected in each frame of the video over six frames. The average flow velocity was obtained from the velocities of the three beads.

#### 2.3.2. Complete Sealing of the Microvalve

Complete sealing of the channel corner was examined by using fluorescent dye. The microvalve filled with water dyed with Rhodamine (AS108, Osaka Sansei, Osaka, Japan) was operated while being observed under the fluorescent microscope (Axiovert 100, Carl Zeiss, Oberkochen, Germany) with a dichroic filter (excitation wavelength 545 nm, emission wavelength 605 nm), and the fluorescent micrograph was obtained using a digital video camera (WRAYCAM-VEX230M, Wraymer, Osaka, Japan). The quantum efficiency of the image sensor at a wavelength of 605 nm is 90%. If the valve is closed completely, all the dyed water at the valve was driven out and the brightness of the image at the entire valve area will be as low as the autofluorescence of the PDMS. The fluorescent micrograph of the operation of the valve filled with water without fluorescent dye was also obtained to measure the autofluorescence of the PDMS. Image processing software (Image J, NIH, Bethesda, MD, USA) was used to generate the difference images between the valve image with and without fluorescent dye to eliminate the effect of autofluorescence.

#### 2.3.3. Response Time of the Microvalve

The response time of the microvalve was measured. Pneumatic pressure of 200 kPa was applied to the pneumatic channel, while the bead suspension flowed in the liquid channel. The flow rate was 5 μL/min. The time between the application of the pneumatic pressure and the closure of the microvalve was measured. The experimental setup was same as that of the experiment in Section 2.3.1. The mean velocity of the microbeads was measured using the same method as the measurement in Section 2.3.1.

## 3. Results

### 3.1. Flow Velocity and Pressure

**Figure 9:**
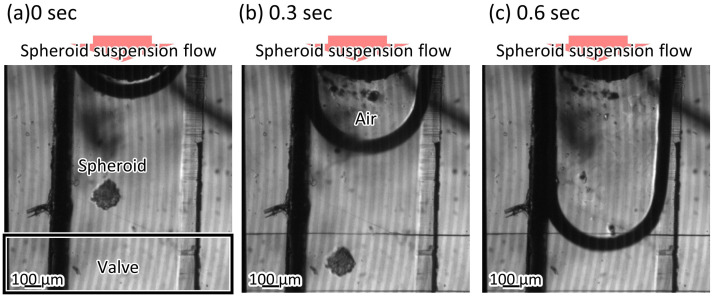
The spheroids passing through the microvalve.

**Figure 10:**
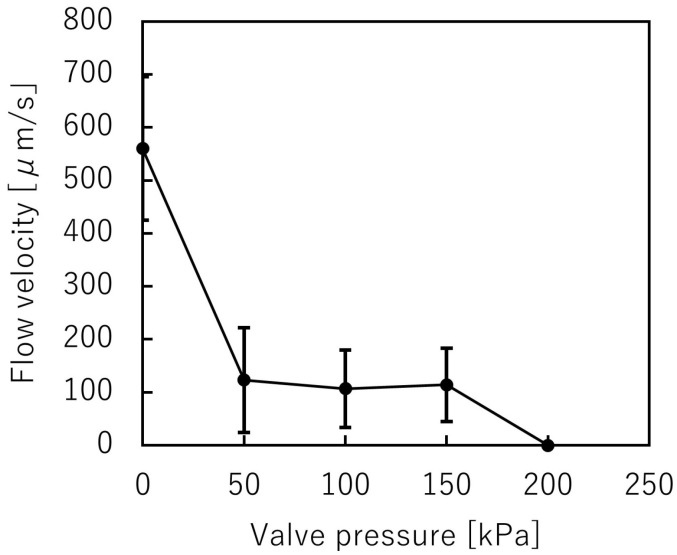
Variations of the mean beads velocity and the pressure applied to the trapezoid-shaped cross-section pneumatic microvalve (*n* = 2).

A suspension including spheroids was introduced into the trapezoid-shaped liquid channel. The depth of the liquid channel was 225 μm, while the spheroids’ diameter was 200 μm. The spheroids could pass through the microvalve without clogging (Figure 9, Video S1), and then the microvalve was used to stop the flow of the suspension. Figure 10 shows the relationship between the applied pressures and the mean velocities of the microbeads flowing in the microchannel. The mean velocities of the flowing microbeads were 656 μm/s, 53 μm/s, 55 μm/s, 65 μm/s, and 0 μm/s, when the applied pressures were 0 kPa, 50 kPa, 100 kPa, 150 kPa, and 200 kPa, respectively. According to the experiment, the microvalve was able to stop the flow of the suspension at 200 kPa in applied pressure.

### 3.2. Complete Sealing of the Microvalve

**Figure 11:**
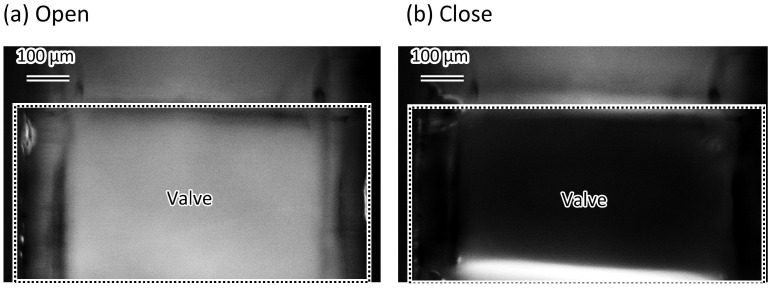
Difference between images of the fluorescent micrograph of the microvalve operation with and without fluorescent dye. (**a**) Corner of the microvalve is filled with fluorescent dyed water when the microvalve is open; (**b**) Fluorescent dyed water at the corner of the microvalve cannot be observed when the microvalve is closed.

Figure 11 shows the difference in the images of the microvalve operation with and without fluorescent dye. The source images of the difference images are shown in Figure S1. The channel corner was filled with the water dyed with Rhodamine when the valve was open (Figure 11a). When the valve was closed, the fluorescence at the channel corner could not be observed (Figure 11b). This indicates that the channel corner is completely closed.

### 3.3. Response Time of the Microvalve

**Figure 12:**
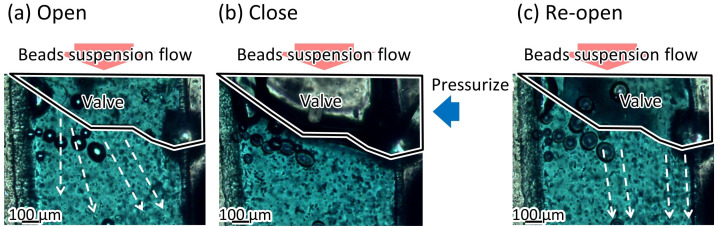
Damming of the beads’ suspension flow using the microvalve. (**a**) Beads in the bead suspension pass through the microvalve when the microvalve is open; (**b**) beads stop when the microvalve is closed; (**c**) beads flow againg when the microvalve is reopened.

**Figure 13:**
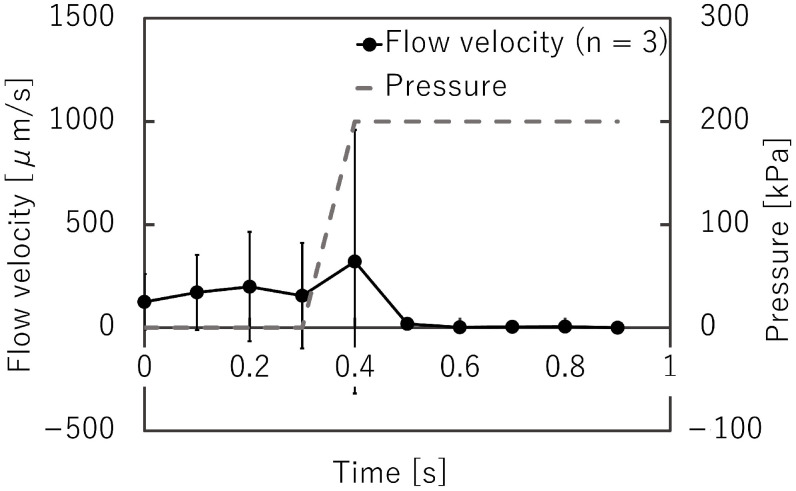
Time variations of the mean bead velocity and pressure applied to the pneumatic microchannel for the microvalve with a liquid microchannel height of 225 μm (*n* = 3).

**Figure 14:**
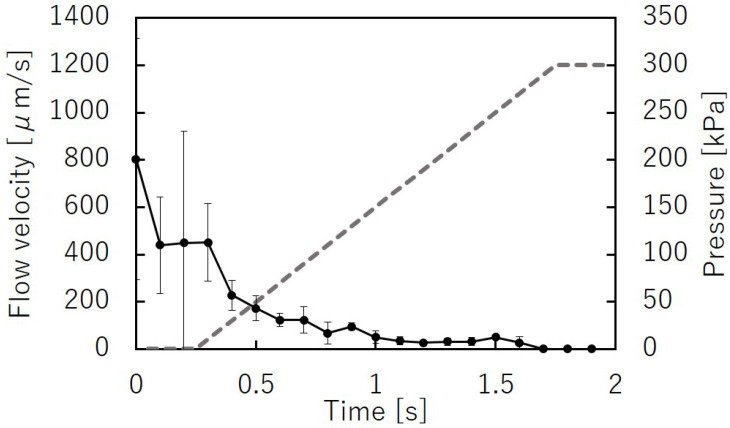
Time variations of the mean bead velocity and pressure applied to the pneumatic microchannel for the microvalve with a liquid microchannel height of 350 μm (*n* = 3).

Figure 12 shows the microbeads flowing in the liquid channel at the valve position. The suspended beads flowed through the liquid microchannel while the microvalve was open (Figure 12a). The flow of the microbeads was stopped when the microvalve was closed (Figure 12b). This indicates that the liquid microchannel, including the inner angles, was completely closed. The beads started to flow again when the microvalve was reopened (Figure 12c). Figure 13 shows the time variations of the mean velocity of the flowing beads and the pressure applied to the pneumatic microchannel (*n* = 3). The microvalve was closed 0.4 s after the pressure of 200 kPa was applied. When the pressure was applied to the balloon, the fluid flowing just below the balloon was pushed out, resulting in faster flow velocity downstream. In this experiment, the flow velocity downstream of the balloon was measured. Due to the pressurization of the balloon, the flow velocity increased temporarily as shown in Figure 13. Video S2 shows the operation of the microvalve. The microvalve with the microchannel of 350 μm depth was also tested. Figure 14 shows the time variations of the mean velocity of the flowing beads and the pressure applied to the pneumatic microchannel. The pressure applied to the pneumatic channel was gradually increased from 0 kPa to 300 kPa to avoid breaking the seal between the membrane and the pneumatic channel. Video S3 shows the operation of the microvalve. The maximum depth of the microchannel valve that could be sealed in this test was 350 μm. The valve could repeatedly open and close 328 times before corruption (S.D. = 15.7, *n* = 3).

## 4. Discussion

In this study, a trapezoid-shaped cross-section valve was developed to close a deep microchannel. The previously developed parallelogram-shaped cross-section valve required 300 kPa of pneumatic pressure to close a microchannel with 100 μm depth, while the trapezoid-shaped cross-section valve required only 200 kPa of pneumatic pressure to close the microchannel with 225 μm depth. This is because the trapezoid-shaped cross-section valve can only close the microchannel by deforming the membrane, whereas the parallelogram valve requires the deformation of the membrane and the overhang at the acute angle. The response time of the trapezoid-shaped cross-section valve was almost identical to that of the parallelogram-shaped cross-section valve (0.35 s). The parameters that affect the response time of the microvalve are the length of the connecting tube and the thickness of the separating membrane. The average response time of the microvalve were 0.31 s (*n* = 3, S.D. = 0.027 s) and 0.36 s (*n* = 3, S.D. = 0.019 s) with lengths of the connecting tube of 55 cm and 110 cm, respectively. The response time was increased by 15.5 % when the length of the connecting tube was increased from 55 cm to 110 cm. There should be some influence of the membrane thickness, since the pressure at which closure begins changes with the thickness of the membrane [26]. Compared with the parallelogram-shaped cross-section valve, the trapezoid-shaped cross-section valve is superior in terms of both closable channel depth and required pressure. The durability of the valve is reasonable because the microvalve in the normal microfludic system does not operate more than 100 times.

Since the trapezoidal cross-sectional channel has an acute angle at the bottom corner, a pressure barrier is created in the hydrophobic channel, preventing the channel edge from being filled with water. In such a case, the pneumatic pressure applied to a droplet will escape from the area not filled with water, which makes it impossible to move the droplet. Although the PDMS used for the microchannel in this study was hydrophobic, it was hydrophilized in order to bond the channel layers. The contact angle was 68.6 degrees and the channel was filled with water up to its edge, and it was confirmed that the slug flow containing spheroids could be moved in the channel in Section 3.1.

It was confirmed that spheroids with 200 μm diameter could pass through the trapezoid-shaped cross-section valve without clogging. Therefore, the valve can accommodate particles with a diameter of approximately 200 μm. The trapezoid-shaped cross-section valve could close the 350 μm high microchannel by gradual pressurization. This suggests that 300 μm particles could be accommodated for applications that do not require switching speed. This would be effective in applications where large particles, such as 3D cultured cells, animalcules, and microcapsules, are handled under precisely controlled conditions.

To develop microvalves to handle particles with diameters over 350 μm, other methods are required. The trapezoid-shaped cross-section cannot close valves deeper than 350 μm. This is because when the pressure larger than 300 kPa is applied to the balloon, it is often burst. If a hard object presses against the fluid channel instead of the inflated balloon, it would be possible to close the channel deeper than 350 μm. The maximum channel depth that normal photolithography can provide is 500 μm. Therefore, microvalves fabricated by machining or 3D printing methods are more suitable when dealing with particles larger than 500 μm.

## 5. Conclusions

A pneumatic microvalve that can control fluid flow through a deep microchannel was developed for handling particles at the scale of hundreds of micrometers. Microbeads and spheroids were used to demonstrate the operation of the proposed microvalve. The flow of 10 μm diameter microbeads through the 225 μm high liquid microchannel was completely stopped when a pressure of 200 kPa was applied to the pneumatic balloon. The channel corner was completely closed. It was also confirmed that a spheroid with a diameter of 200 μm could pass through without clogging when the pressure applied was 0 kPa. The trapezoid-shaped cross-section with a depth of 350 μm could be closed by gradually applying a pressure of 300 kPa. In future, we will integrate the microvalve into microfluidic devices for practical applications.

## Data Availability

The data that support the findings of this study are available from the corresponding author upon reasonable request.

## Supplementary Materials

The following are available online at https://www.mdpi.com/article/10.3390/mi12111403/s1, Video S1: Motion of a spheroid passing though the microvalve with a liquid microchannel of depth 225 μm, membrane thickness 40 μm, and θ = 60 degrees. Video S2: Motion of the bead suspension passing though the microvalve with a liquid microchannel of depth 225 μm, membrane thickness 40 μm, and θ = 60 degrees. Video S3: Motion of the bead suspension passing though the microvalve with a liquid microchannel of depth 350 μm, membrane thickness 40 μm, and θ = 60 degrees. Figure S1: The source images of the difference images shown in Figure 11.
