# Supplementary figures and images for: Microvalve with Trapezoid-Shaped Cross-Section for Deep Microchannels
Source: Micromachines (Basel). 2021 Nov 15;12(11):1403. doi: 10.3390/mi12111403 (PMC8622413; doi:10.3390/mi12111403)

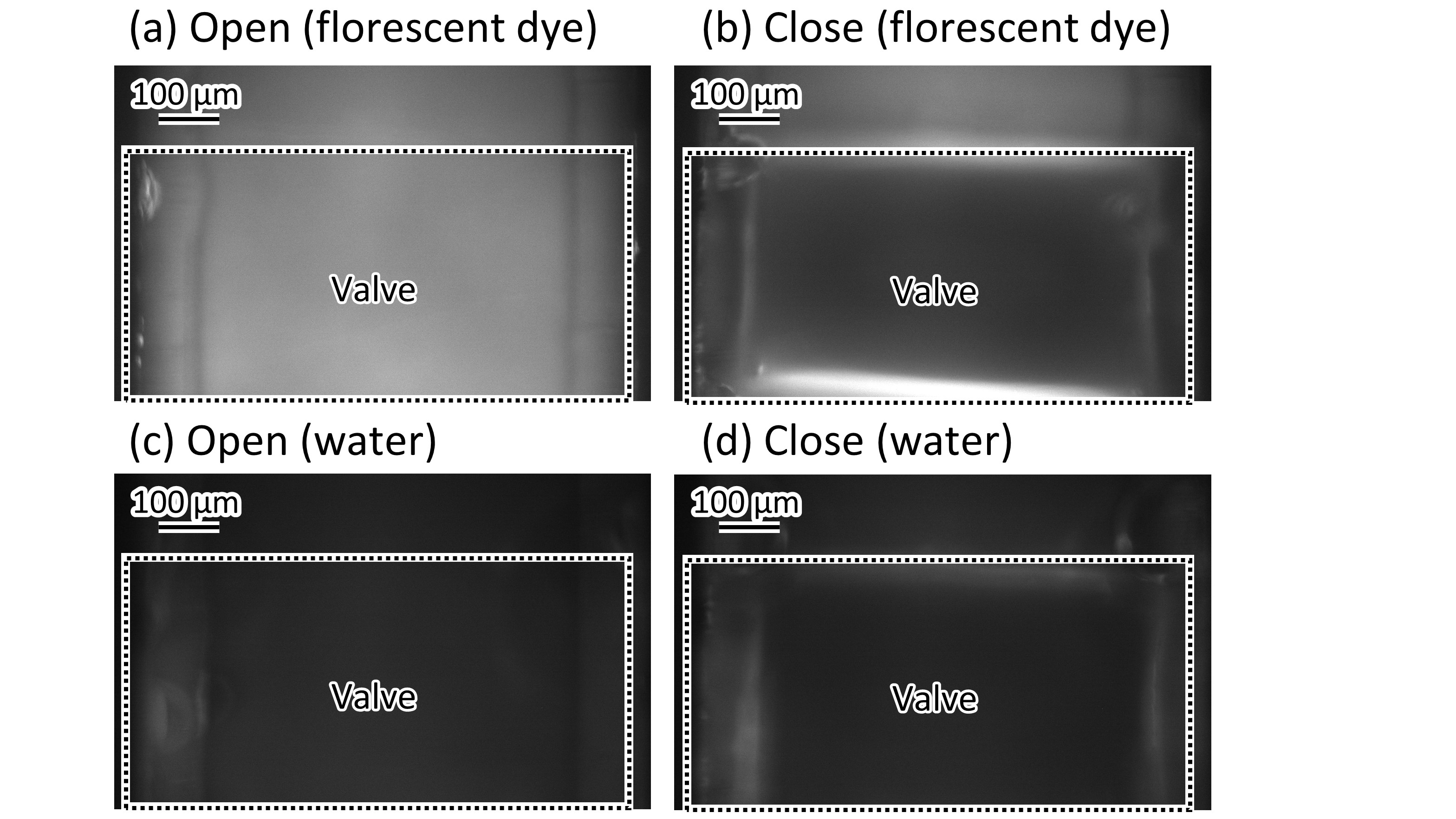

Supplement: Supplementary file 1 [file micromachines-12-01403-s001.zip › Figure S1.JPG.jpg]
